# Contrasts in Oxidative Potential and Other Particulate Matter Characteristics Collected Near Major Streets and Background Locations

**DOI:** 10.1289/ehp.1103667

**Published:** 2011-10-20

**Authors:** Hanna Boogaard, Nicole A.H. Janssen, Paul H. Fischer, Gerard P.A. Kos, Ernie P. Weijers, Flemming R. Cassee, Saskia C. van der Zee, Jeroen J. de Hartog, Bert Brunekreef, Gerard Hoek

**Affiliations:** 1 Institute for Risk Assessment Sciences, Division Environmental Epidemiology, Utrecht University, Utrecht, the Netherlands; 2 National Institute for Public Health and the Environment, Centre for Environmental Health, Bilthoven, the Netherlands; 3 Energy Research Centre of the Netherlands, Department of Air Quality, Petten, the Netherlands; 4 Municipal Health Service Amsterdam, Department of Environmental Health, Amsterdam, the Netherlands; 5 Julius Center for Health Sciences and Primary Care, University Medical Center Utrecht, Utrecht, the Netherlands

**Keywords:** background locations, metals, oxidative potential, particulate matter, urban streets

## Abstract

Background: Measuring the oxidative potential of airborne particulate matter (PM) may provide a more health-based exposure measure by integrating various biologically relevant properties of PM into a single predictor of biological activity.

Objectives: We aimed to assess the contrast in oxidative potential of PM collected at major urban streets and background locations, the associaton of oxidative potential with other PM characteristics, and the oxidative potential in different PM size fractions.

Methods: Measurements of PM with aerodynamic diameter ≤ 10 μm (PM_10_), PM with aerodynamic diameter ≤ 2.5 μm (PM_2.5_), soot, elemental composition, and oxidative potential of PM were conducted simultaneously in samples from 8 major streets and 10 urban and suburban background locations in the Netherlands. Six 1-week measurements were performed at each location over a 6-month period in 2008. Oxidative potential was measured as the ability to generate hydroxyl radicals in the presence of hydrogen peroxide in all PM_10_ samples and a subset of PM_2.5_ samples.

Results: The PM_10_ oxidative potential of samples from major streets was 3.6 times higher than at urban background locations, exceeding the contrast for PM mass, soot, and all measured chemical PM characteristics. The contrast between major streets and suburban background locations was even higher (factor of 6.5). Oxidative potential was highly correlated with soot, barium, chromium, copper, iron, and manganese. Oxidative potential of PM_10_ was 4.6 times higher than the oxidative potential of PM_2.5_ when expressed per volume unit and 3.1 times higher when expressed per mass unit.

Conclusions: The oxidative potential of PM near major urban roads was highly elevated compared with urban and suburban background locations, and the contrast was greater than that for any other measured PM characteristic.

Numerous epidemiological studies have documented the effects of particulate matter (PM) with aerodynamic diameter ≤ 10 μm (PM_10_) and with aerodynamic diameter ≤ 2.5 μm (PM_2.5_) air pollution on morbidity and mortality from respiratory and cardiovascular diseases [Health Effects Institute (HEI) 2010]. Although studies often have measured PM concentrations on a mass basis, proximity to traffic is much better reflected by other PM characteristics, such as soot or particle number concentrations, than by PM mass levels (Fischer et al. 2000; Harrison et al. 2004). In addition, characteristics such as surface area, acidity, and particle composition (including transition metals and hydrocarbons content) are likely to affect PM toxicity (Donaldson et al. 2005; Knaapen et al. 2004; Nel 2005). Oxidative stress has been suggested as an important underlying mechanism of action by which exposure to PM may lead to adverse health effects (Nel 2005). Oxidative stress results when the generation of reactive oxygen species (ROS), or free radicals, exceeds the available antioxidant defenses. ROS can damage membrane lipids, proteins, and DNA, which can result in cell death via either necrotic or apoptotic processes (HEI 2010; Nel 2005). High levels of oxidative stress lead to inflammatory response via activation of various transcription factors and stimulation of cytokine production (Donaldson et al. 2005; Nel 2005). Inflammation plays an important role in respiratory and cardiovascular diseases that have been associated with PM.

Oxidative potential of PM integrates various biologically relevant properties, including size, surface, and chemical composition of PM. Therefore, it may provide a more health-based exposure measure than PM mass alone and may be a better measure of the biologically effective dose that drives adverse health effects (Borm et al. 2007). There is no consensus regarding the most appropriate assay to measure the oxidative potential of PM (Ayres et al. 2008). Studies have made use of various assays, including the ability of PM to induce hydroxyl radicals (^_•_^OH) (Shi et al. 2003a, 2003b), the ability of PM to deplete antioxidants (Mudway et al. 2004), and the promotion of electron transfer measured by the consumption of dithiothreitol (DTT) (Biswas et al. 2009; Cho et al. 2005).

Several studies have shown increased cardiorespiratory morbidity and mortality related to living near major roadways (HEI 2010). Candidate pollutants that may be responsible include ultrafine particles [PM with aerodynamic diameter ≤ 0.1 μm (PM_0.1_)], soot, and polycyclic aromatic hydrocarbons, partly based on the large contrast in concentration (Hoek et al. 2010). A more biologically relevant measure such as the oxidative potential may help in the interpretation of these studies. To our knowledge, no studies have included a systematic comparison of oxidative potential between PM collected near major urban streets and background locations. A few studies have compared the oxidative potential of PM and other PM characteristics. Künzli et al. (2006) compared oxidative potential with other PM_2.5_ characteristics measured at 20 different European locations and reported that oxidative potential varied by one order of magnitude both in time and in space, and that it was not well correlated with other PM characteristics. Other studies on the oxidative potential of outdoor PM have focused on the potential for PM to generate oxidative stress (Vidrio et al. 2009) and on the difference in oxidative potential between different PM fractions or PM sources (Cho et al. 2005; Mudway et al. 2004; Prahalad et al. 1999; Shi et al. 2003a; Verma et al. 2009).

Previously we reported a substantial contrast between moderately busy streets and background locations for elemental concentrations of chromium (Cr), copper (Cu), and iron (Fe) (factor 2 to 3), and for soot and PM_0.1_ (factor 1.9), whereas the contrast was small for PM_10_ and PM_2.5_ mass concentrations (factor 1.2) (Boogaard, et al, 2011).Because the chemical analyses conducted did not provide information regarding bioavailability of elements, we also aimed to evaluate whether these chemical differences were found in oxidative potential.

The present study was conducted within the framework of a study to evaluate the air quality and health benefits of low emission zones directed at heavy-duty vehicles in several Dutch cities. Our study includes comprehensive measurements of air quality and of the health status of populations within the study areas before and 2 years after the policy was implemented (Boogaard et al. 2011). Here we report the oxidative potential of PM measurements at major urban streets and urban and suburban background locations collected at baseline in 2008. We aimed to assess the contrast in oxidative potential of PM collected at major urban streets and background locations, the association of oxidative potential with other PM characteristics, and the oxidative potential in different PM size fractions.

## Materials and Methods

*Air sample collection.* PM_10_, PM_2.5_, soot, elemental composition, and oxidative potential of PM were measured in samples collected at 18 locations in the Netherlands: eight major streets in five cities, five urban background locations (one in each city), four suburban background locations (one of which was used as a comparison location for two nearby cities), and one urban background location in the center of the Netherlands where continuous measurements were made throughout the study period.

At each location, six weekly samples were collected over a 6-month period beginning in June 2008 (Boogaard et al. 2011). Each street and its corresponding urban and suburban background location were measured simultaneously during the same week. For budgetary reasons, locations were measured in two rounds, with two cities included in one round and the remaining three in the other [for the exact air sampling schedule, see Supplemental Material, Table 1 (http://dx.doi.org/10.1289/ehp.1103667)]. As noted above, continuous measurements were made during the whole study period at a central urban background location, which were used to adjust discontinuous measurements at the other locations to account for temporal variations in background concentrations. The eight street locations had a traffic intensity varying between approximately 10,000 and 19,000 motor vehicles passing every 24 hr (for additional details regarding the urban street locations, see Supplemental Material, Table 2). The five urban background locations were located in the city centers but not in the direct vicinity of major streets. The four suburban background locations were in villages near the selected cities.

**Table 1:** Median correlation between PM characteristics measured simultaneously at the central urban background location and other locations.

Location	*n*	^•^OH	PM_10_	PM_2.5_	Soot	Cu	Fe	S
Suburban background locations		4		0.98		0.97		0.97		0.97		0.93		0.73		0.97
Urban background locations		5		0.81		0.92		0.99		0.97		0.90		0.91		0.94
Urban street locations		8		0.61		0.92		0.95		0.72		0.40		0.46		0.94
All locations		17		0.81		0.92		0.97		0.90		0.85		0.58		0.94
Pearson correlation coefficients were first calculated among samples collected at each location separately (*n* = 4–6 measurements per location) and then summarized with the use of the median, either by site type (suburban, urban, and major urban street) or all locations together.																

**Table 2:** Median PM characteristics at the different urban streets and median ratio between street and corresponding urban background.

City	Urban street*a*	^•^OH*b*	PM_10_ (μg/m^3^)	PM_2.5_ (μg/m^3^)	Soot (10^–5^/m)	Cr (ng/m^3^)	Cu (ng/m^3^)	Fe (ng/m^3^)
Amsterdam		Haarlemmerweg		50,200		28.6		19.1		3.9		8.9		63.0		1205.7
				6.8		1.2		1.2		2.1		3.5		5.1		3.4
Amsterdam		Hoofdweg		23,500		24.1		15.0		2.8		5.9		27.1		606.7
				2.6		1.1		1.0		1.7		2.0		2.4		1.8
The Hague		Stille Veerkade		38,600		32.5		17.2		4.3		9.8		52.8		1180.6
				4.2		1.3		1.2		2.7		2.9		3.6		2.8
Den Bosch		Brugstraat		49,500		32.7		18.1		3.6		7.5		45.9		975.2
				4.0		1.3		1.3		2.1		2.4		3.6		2.9
Den Bosch		Koningsweg		38,900		30.0		17.5		3.0		5.9		35.1		750.4
				2.5		1.1		1.2		1.9		1.5		2.1		2.0
Tilburg		HVB		24,000		31.5		17.5		2.5		6.0		35.0		666.1
				3.5		1.1		1.1		1.5		2.2		2.6		2.6
Utrecht		Vleutenseweg		18,500		25.0		15.5		2.1		4.8		20.6		387.8
				1.6		1.0		1.1		1.5		1.3		1.5		1.1
Utrecht		Weerdsingel Wz		48,100		28.2		17.2		3.6		7.1		42.0		971.0
				4.1		1.2		1.1		2.2		2.3		3.1		2.7
Overall ratio				3.6		1.2		1.2		1.9		2.2		2.8		2.5
*p*-Value				< 0.0001		< 0.0001		< 0.0001		< 0.0001		< 0.0001		< 0.0001		< 0.0001
HVB, Hart van Brabantlaan. Data are median concentrations and median ratios with corresponding urban background. Concentrations of elements are slightly different from those published previously (Boogaard et al. 2011), because median values are presented and adjusted for temporal variation using data from the central reference location. **a**Streets in Amsterdam and The Hague and corresponding background locations were measured during the same weeks. Den Bosch, Tilburg, and Utrecht (street and background locations) were measured simultaneously during a different set of weeks. **b**Oxidative potential of PM_10_ is given as arbitrary units per cubic meter of air.																

*Elemental composition and other PM characteristics.* Exact methods and results of the analysis of elemental composition and other PM characteristics have been published previously (Boogaard et al. 2011). Briefly, PM_10_ and PM_2.5_ were collected gravimetrically on Teflon filters using PM_10_ personal samplers (MSP Corp., Shoreview, MN, USA) and PM_2.5_ GK2.05 cyclones (BGI Inc., Waltham, MA, USA) at all locations. Soot content of all PM_10_ filters was measured using a Smoke Stain Reflectometer (model M43D; Diffusion Systems, London, UK) and transformed into absorption coefficients. In total, 105 (92%) PM_10_ filters and 104 (91%) PM_2.5_ filters were available for analysis. For quality assurance, 12 field blank filters and 19 duplicate measurements also were collected. All measurements were above the detection limit (LOD), with a coefficient of variation (CV; a measure of precision) < 10%. All filters were analyzed with energy dispersive X-ray fluorescence spectrometry (ED-XRF) at Cooper Environmental Services (Portland, OR, USA). In this article we report elemental concentrations derived from PM_10_ filters. All elements were above the LOD in all samples except for aluminum (Al), barium (Ba), and vanadium (V) (72–99% of samples > LOD). The CVs for duplicate measurements were < 25%.

*Oxidative potential.* The oxidative potential of PM was measured with the electron paramagnetic resonance (EPR) assay, which uses conditions similar to those in the lungs (Borm et al. 2007; Shi et al. 2003b). Besides the EPR assay, other acellular assays exist, such as the DTT assay and the antioxidant depletion assay, which are sensitive to slightly different PM characteristics (Ayres et al. 2008). We chose the EPR assay because it requires relatively little material, its costs are relatively low, and it has been used in other large air pollution studies (e.g., Künzli et al. 2006).

After ED-XRF analyses, oxidative potential was assessed in all PM_10_ samples and a subset of PM_2.5_ samples (because of budget restraints) in the laboratory of the Province of Limburg, the Netherlands. All PM_2.5_ samples from two sampling weeks in October and December 2008 were included. To our knowledge, effects of XRF analysis on oxidative potential have not been investigated, but XRF analyses will likely not change the composition or valence state of the material on the samples and is therefore not expected to affect oxidative potential.

The EPR assay measures oxidative potential based on the ability of PM to generate ^_•_^OH in the presence of hydrogen peroxide (H_2_O_2_), as described in detail elsewhere (Künzli et al. 2006; Shi et al. 2003b). Briefly, PM suspensions were prepared from the Teflon filters. Laboratory blank filters were treated similarly and used as controls in the experiments. Generation of ^_•_^OH by particle suspensions was studied in the presence of H_2_O_2_ and the spin-trap 5,5-dimethyl-1-pyrroline-*N*-oxide (DMPO). For the ^_•_^OH analyses, 50 μL of the particle suspension was mixed with 50 μL H_2_O_2_ and 100 μL DMPO. The mixture was incubated in the dark and shaken continuously at 37°C before being filtered through a 0.1-μm pore filter. The clear filtrate was measured with a Miniscope MS100 EPR spectrometer, (Magnettech, Berlin, Germany) all under standard conditions. The oxidative potential of each PM sample was calculated from the sum of total amplitudes of the DMPO–^_•_^OH quartet signal and expressed as the total amplitude in arbitrary units divided by the sampled air volume (cubic meters). Unless stated otherwise, ^_•_^OH is expressed per volume of air, because this reflects exposure more as encountered by the airways under real-life conditions compared to expression per milligram of PM. Mean field blank readings (mean, 704; range 377–1,063, all in arbitrary units per cubic meters) were subtracted from all measurements. The average ratio of the blank readings to the samples was 0.09. All but one oxidative potential measurement were above the LOD (> 725/m^3^), calculated as three times the standard deviation of the field blank readings. The one value below the LOD was retained. The CV of 10 oxidative potential duplicate measurements was 19%.

*Data management and analysis.* We adjusted our measurements for temporal variation using the central reference location. Specifically, we determined the difference between the average value measured over the 6-month study period at the central reference location and the value measured during each sampling week at the same location, and then added or subtracted this value from the observed measurements for samples from the other locations during corresponding weeks (Hoek et al. 2002). Because this procedure has been applied in previous studies to PM, soot, and nitrogen dioxide, but not elemental composition and oxidative potential, we first compared how measurements of the central reference location correlated with other locations, measured simultaneously. Pearson correlation coefficients were first calculated among samples collected at each location separately and then summarized by site type (suburban, urban, and major urban street) with the use of the median. Correlation coefficients from an individual location had limited precision because they were based on at most six data points.

*Spatial variation.* We calculated ratios between major streets and matching background locations for the different PM characteristics. Median ratios were reported per major street (*n* = 6). In addition, an overall median ratio was given (*n* = 48). We calculated the median because it is less influenced by outlier values. The overall median ratio was tested for statistical significance with the nonparametric signed rank test.

*Association between oxidative potential and other PM characteristics.* Pearson correlation coefficients were calculated between location-specific median and averages of the PM characteristics as well as for the individual measurements. Scatterplots were made, and Cooks distance was calculated to investigate the extent to which the correlation analyses were driven by outliers.

To investigate which PM_10_ characteristics were associated with oxidative potential variability, we first used univariate mixed models based on individual measurements, using the natural logarithm of oxidative potential as the dependent variable. We identified the PM characteristic that best predicted oxidative potential by comparing the Akaike information criterion (AIC) values of the univariate models, and then added other PM characteristics (with *p* < 0.10) to that model to determine if they reduced the AIC further (i.e., improve model fit).

## Results

**Figure 1:**
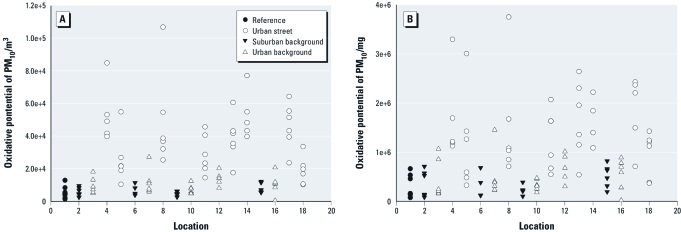
Oxidative potential of PM_10_ measurements (*A*, per cubic meter; *B*, per milligram) per location (*n* = 18, four to six samples per location). Locations: 1, central reference location (Utrecht University); 2, suburban Amsterdam; 3, urban background, Amsterdam; 4 and 5, urban streets, Amsterdam; 6, suburban The Hague; 7, urban background, The Hague; 8, urban street, The Hague; 9, suburban Den Bosch + Tilburg; 10, urban background, Tilburg; 11, urban street, Tilburg; 12, urban background, Den Bosch; 13 and 14, urban streets, Den Bosch; 15, suburban Utrecht; 16, urban background, Utrecht; 17 and 18, urban streets, Utrecht.

*Temporal variation.* High temporal variability in the oxidative potential of individual PM_10_ samples for all locations is evident when expressed per cubic meter (Figure 1A). This variability is not explained by the variability in absolute particle mass collected, because the oxidative potential expressed per milligram showed a similar pattern (Figure 1B).

The oxidative potential of samples collected at the urban and suburban background locations was highly correlated with simultaneously measured values for samples collected at the central urban background reference location (median correlations of 0.81 and 0.98, respectively; Table 1). Median correlations between urban and suburban locations and the central reference location for corresponding weeks were also high for PM mass and other PM characteristics. In contrast, median correlations between measurements at the street locations and the central reference location were lower for oxidative potential (*R* = 0.61) and other traffic-related indicators such as soot, Cu, and Fe, but not for PM_2.5_ and sulfur (S), which are driven primarily by long-range transport of PM from other sources.

*Spatial variation.* Oxidative potential of PM_10_ was on average 3.6 times higher at the street locations than at the corresponding urban background locations in each city (Table 2). This contrast was larger than observed for the transition metals Fe and Cu (2.5 and 2.8 times higher, respectively) or for soot and PM_0.1_ (1.9 times higher for both). There was relatively little spatial contrast in the regulated PM mass metrics PM_10_ and PM_2.5_ (ratio of 1.2 for both).

The highest oxidative potential contrasts were found for two streets that were classified as street canyons (Stille Veerkade and Brugstraat, which are narrow streets with adjoining high buildings on both sides) and two streets with buildings one side of the street only (Haarlemmerweg and Weerdsingel Wz; Table 2), consistent with the higher soot, Cr, Cu, and Fe contrasts previously reported for the same streets (Boogaard et al. 2011).

The contrast in oxidative potential between matched urban and suburban locations was also substantial (1.8 times higher at the urban locations). This oxidative potential of PM_10_ from the major streets was 6.5 times higher than that of the matched suburban locations [for median ratios streets and urban background locations vs. suburban background locations, see Supplemental Material, Table 3 (http://dx.doi.org/10.1289/ehp.1103667)].

**Table 3:** Pearson correlation coefficients between oxidative potential (^•^OH) of PM_10_ and other PM characteristics.

PM characteristic	^•^OH	*p*-Value
PM_10_		0.20		0.05
PM_2.5_		0.08		0.43
Soot		0.73		< 0.0001
Al		0.12		0.21
Ba		0.58		< 0.0001
Br		–0.01		0.88
Ca		0.22		0.02
Cl		0.07		0.46
Cr		0.73		< 0.0001
Cu		0.89		< 0.0001
Fe		0.84		< 0.0001
Mn		0.61		< 0.0001
Ni		0.22		0.02
Pb		–0.01		0.89
S		–0.04		0.64
Si		0.19		0.05
Ti		0.15		0.13
V		0.01		0.90
Zn		0.30		0.002
Abbreviations: Br, bromine; Ca, calcium; Zn, zinc. Numbers of observations vary between 97 (^•^OH and PM_2.5_) and 105 (all others). Elements were derived from PM_10_ filters.				

**Figure 2:**
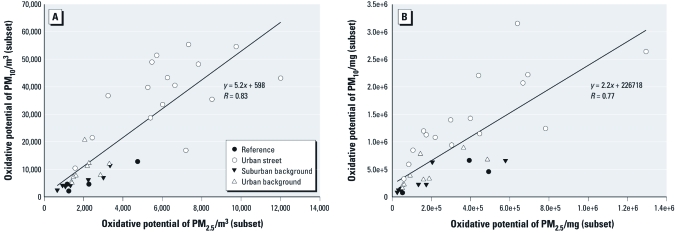
Association between oxidative potential of PM_2.5_ and PM_10_ samples measured simultaneously (*n* = 36).

*Oxidative potential of different PM size fractions.* The oxidative potential of PM_10_ was 4.6 times higher than the oxidative potential of PM_2.5_ measured simultaneously in a subset of filters when expressed per volume unit (Figure 2A) and 3.1 times higher when expressed per mass unit (Figure 2B) across all samples tested (regardless of location or sampling week). These median ratios were statistically significantly different from unity (*p* < 0.0001). Note that the model equations in Figure 2 described average ratios together with estimated intercepts. The correlations between the oxidative potentials of PM_10_ and PM_2.5_ were 0.83 and 0.77 when expressed per volume and mass unit, respectively.

**Figure 3:**
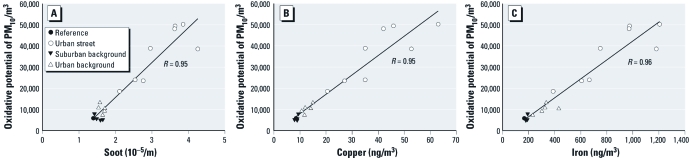
Associations of median oxidative potential of PM_10_ and soot (*A*), Cu (*B*), and Fe (*C*) concentrations per location,**corrected for temporal variation using data from the central reference location.

*Association between oxidative potential and other PM characteristics.* High correlations (*R* > 0.80) were found between median and average oxidative potential, and soot, Ba, Cr, Cu, Fe, and manganese (Mn), in the PM_10_ fraction for the 17 locations (see Figure 3 for median values of soot, Cu, and Fe per location). Further analyses were hampered by the small sample size.

Across all individual samples (regardless of location or sampling week), oxidative potential was highly correlated with soot, Ba, Cr, Cu, Fe, and Mn (Table 3). After excluding outlier values (Cooks distance > 1), correlations did not change substantially. PM_10_ and PM_2.5_ measurements were not significantly associated with oxidative potential. Results were comparable when we limited the comparisons to samples collected during the same sampling weeks (data not shown).

When we analyzed the oxidative potential of PM_10_ using mixed models based on individual measurements with location treated as a random effect, the amount of Cu in PM_10_ explained most of the variability in oxidative potential in a one-pollutant model [for the exact results of the mixed model analysis, see Supplemental Material, Table 4 (http://dx.doi.org/10.1289/ehp.1103667)]. A two-pollutant model that included Cu and PM_10_ mass had a smaller AIC than the model with Cu alone. In this two-pollutant model, an interquartile range (IQR) increase in Cu (22 ng/m^3^) was associated with a 176% increase in oxidative potential, whereas an IQR increase in PM_10_ (13 μg/m^3^) was associated with a 35% decrease in oxidative potential. Because the correlation between Cu and PM_10_ was 0.51, the negative PM_10_ slope is unlikely because of collinearity. Cu was highly correlated with soot (*R* = 0.90), Ba (*R* = 0.64), Cr (*R* = 0.84), Fe (*R* = 0.95), and Mn (*R* = 0.74), so it was not possible to estimate the independent associations of these PM characteristics with the oxidative potential of PM_10_.

## Discussion

The oxidative potential of PM_10_ collected at 18 locations spread over the Netherlands varied substantially in time and space. On average, the oxidative potential of PM collected at major urban streets (expressed per volume unit) was 3.6 times higher than simultaneously measured PM at urban background locations. This exceeded contrasts for other PM characteristics such as soot and transition metal contents. The oxidative potential contrast between measurements collected near major urban streets and suburban background locations was even higher (factor 6.5 difference). The oxidative potential of PM_10_ was 4.6 times greater than that of PM_2.5_ measured simultaneously in a subset of filters when expressed per volume unit, and 3.1 times greater when expressed per mass unit. Oxidative potential was highly correlated with soot, Ba, Cr, Cu, Fe, and Mn.

*Spatial variation.* Previously we reported substantially higher concentrations of soot, ultrafine particles, and the transition metals Cr, Cu, and Fe at the same moderately busy streets compared with background locations (Boogaard et al. 2011). This reflects local exhaust emissions as well as nonexhaust traffic emissions such as Cu and Fe from brake and tire wear. The present article shows that this contrast is even higher for oxidative potential. This is important, because there is growing evidence that the ability of PM to cause oxidative stress may be an important mechanism for the adverse health effects of particles (Ayres et al. 2008; HEI 2010).

We used the EPR assay to characterize the oxidative potential of PM. The EPR assay is especially sensitive to transition metals driving ^_•_^OH generation mechanisms via the Fenton reaction, which involves the reduction of H_2_O_2_ by a transition metal ion (Shi et al. 2006). Although transition metals influence oxidative potential as measured by the EPR assay, the finding of a high contrast in total transition metal concentrations obtained by ED-XRF does not necessarily imply a high contrast in oxidative potential because ED-XRF measurements do not reflect the valence state or bioavailability of the metals, which can influence oxidative potential substantially. Shi et al. (2003a) showed a high ability for Cu(II), V(II), V(V), and Fe(II) and less ability for Fe(III) and nickel-II [Ni(II)] to generate oxidative potential of PM.

The contrast in oxidative potential of PM from major urban streets and urban background locations may have been greater than corresponding contrasts in the measured concentrations of transition metals because of differences in the concentrations of bioavailable (i.e., soluble) metals between the two site types. Alternatively, other unmeasured pollutants near major roads may affect oxidative potential, for example, ultrafine particles. Some studies have shown that ultrafine particles have higher oxidant potential than do larger PM fractions (Araujo et al. 2008; Cho et al. 2005; Li et al. 2003). These studies used the DTT assay to measure oxidative potential, which is more sensitive to organic compounds that are concentrated in the ultrafine fraction of PM than is the EPR assay, which is driven by chemical composition rather than by particle size. However, in a subset of two streets (Haarlemmerweg, Vleutenseweg), ultrafine particles showed a smaller contrast at urban streets compared with matching suburban locations than for Cr, Cu, and Fe (Boogaard et al. 2011). Finally, it is also possible that differences in oxidative potential of PM from different locations may reflect additive or synergistic effects of different components of PM (Wilson et al. 2002).

Only a few field studies have systematically compared oxidative potential of PM from different locations (Künzli et al. 2006; Shi et al. 2006), and none have compared oxidative potential of PM from major urban streets with urban background locations. A study in the western part of Germany found approximately 1.7 times higher ^_•_^OH generation in PM from three urban locations compared with a more rural location (Shi et al. 2006), which is very close to the contrast we found between urban and suburban background locations (1.8 times higher for urban vs. suburban). Künzli et al. (2006) reported a 9.6-fold contrast in mean oxidative potential measured as ^_•_^OH generation between the measured European cities. Most urban sites can be classified as urban background locations. The mean oxidative potential for the second lowest and second highest cities differed by a factor of 3.1, very close to the variability in our study, which covered a much smaller geographic area. This documents that oxidative potential varies both on a large and on a fine spatial scale.

*Association between oxidative potential and other PM characteristics.* We reported high correlations between the oxidative potential of PM_10_ and the concentration of the transition metals Ba, Cr, Cu, Fe, Mn, and soot in the PM_10_ fraction. This is consistent with previous observations that transition metals drive ^_•_^OH generation via the Fenton reaction, which involves the reduction of H_2_O_2_ by a transition metal ion (Shi et al. 2006). High correlations between oxidative potential and transition metals such as Cu and Fe have been reported previously based on different assays (Briedé et al. 2005; Mudway et al. 2004; Shi et al. 2006). The correlations between oxidative potential and transition metals in our study (e.g., 0.89 and 0.84 for Cu and Fe, respectively) were higher than in the Künzli et al. (2006) study (0.39 and 0.45 for Cu and Fe, respectively), possibly because of the greater diversity of locations included in that study. In their study, transition metal concentrations were also measured with ED-XRF. Differences between studies may reflect differences in the bioavailability of the metals for Fenton reactions.

In the present study, the amount of Cu explained variability in oxidative potential of PM_10_ best in univariate mixed models. However, because of the high correlation between Cu and soot, Ba, Cr, Fe, and Mn, we could not further disentangle independent contributions. Nawrot et al. (2009) investigated the oxidative potential of PM_2.5_ in relation to other PM characteristics using the same data as Künzli et al. (2006). In their study, 716 filters were available for analysis from 20 different locations that were each sampled over at least 12 months. Across the 20 locations, elements that explained most of the variation in oxidative potential were Fe (positive), chlorine (Cl), and sodium (Na; inverse) (Nawrot et al. 2009). In our study, Fe was also significantly and positively correlated with oxidative potential of PM_10_, whereas Cl and Na concentrations were not related to oxidative potential (data not shown for Na). In 15 locations Nawrot et al. (2009) showed a positive relation between oxidative potential and one of more of the measured transition metals [Cu, Fe, Mn, lead (Pb), titanium (Ti), and V]. Associations between PM_2.5_ mass concentrations and oxidative potential varied in that study, with positive associations in 8 locations, no association in 10 locations, and inverse associations in 2 locations. We found an inverse relation between oxidative potential and PM_10_ mass in a model that included Cu. An effect of sulfate—a major component of PM mass—may partly explain this relation, because sulfate is able to modify transition-metal–catalyzed oxidative reactions by scavenging ^_•_^OH to yield less reactive inorganic radicals (De Laat et al. 2004).

We also found a positive relationship between oxidative potential and soot. Künzli et al. (2006) reported a much lower correlation between annual average ^_•_^OH and soot (*R* = 0.16, compared with 0.73 in our study). Their study was not designed to look at traffic pollution effects specifically, because most of the sites in that study are urban background locations. Temporal correlations between ^_•_^OH and soot were low to moderate as well, with the highest correlation among samples collected in Reykjavik, Iceland (0.50). Correlation between soot and oxidative potential determined with the DTT assay in another study was also high (*R* = 0.89) (Cho et al. 2005).

*Oxidative potential of different PM size fractions.* We found a significantly higher oxidative potential in PM_10_ than in PM_2.5_ samples compared on both an equal volume and an equal mass basis. This comparison was based on a subset of the data (36%), and the association might have been different if it had been assessed in all study samples. The average PM_10_ oxidative potential for samples collected in the 2 weeks when oxidative potential was measured in PM_2.5_ as well was 20,900/m^3^, which is similar to the average value of 21,800/m^3^ for the other four measurement weeks. The ratio of PM_2.5_ to PM_10_ mass was somewhat higher for the 2-week subset than for the other measurement weeks (0.67 compared with 0.53), mostly due to higher S and silicon (Si) contributions to PM_2.5_ mass. The overall ratio of Cu in PM_2.5_ and Cu in PM_10_ was 0.16 for the selected period and 0.15 for the nonselected period. For Fe these ratios were 0.22 and 0.18. Therefore, it seems unlikely that the differences in the oxidative potential of PM_10_ and PM_2.5_ would have varied substantially if all samples had been measured.

It is likely that chemical composition, rather than PM size, is responsible for differences in the oxidative potential of PM_10_ and PM_2.5_. Total transition metals were especially present in the coarse fraction of PM_10_ (≥ 2.5 μm). Other studies have also reported that coarse particles rather than fine (PM_2.5_) particles had the highest oxidative potential on a per unit mass basis (Shi et al. 2003a, 2006). Differences between studies are partly related to the assay used: the EPR assay used in the present study being more sensitive to transition metals, whereas the DTT assay used by others is more sensitive to organic components such as polycyclic aromatic hydrocarbons and quinones. The high oxidative potential of coarse particles based on the EPR assay supports the hypothesis that coarse particles may be able to induce oxidative stress, and that ultimately this may lead to adverse health effects. Epidemiological studies have found associations between daily variation in coarse particle concentrations and a range of adverse respiratory and cardiovascular health effects (Brunekreef and Forsberg. 2005). Our study adds to the evidence that these effects may be due to similar mechanisms as hypothesized for fine and ultrafine particles.

*Limitations.* An important limitation is that we used only one assay to measure oxidative potential of PM. How the EPR assay relates to oxidative potential as measured by other assays is not clear. All laboratory tests of oxidative potential are sensitive to slightly different panels of metals or organic PM characteristics such as quinones and endotoxins (Ayres et al. 2008). The EPR assay used here is not sensitive to quinones or endotoxins, in contrast with assays such as the antioxidant depletion assay (Kelly 2003). Künzli et al. (2006) used two different assays to measure oxidative potential of PM: the EPR and the antioxidant depletion assay. The correlation between the EPR and the antioxidant depletion assay results varied between 0.18 and 0.65 for the different cities, showing poor to only moderate agreement between assays.

Like most other studies investigating oxidative potential of PM, PM was first collected on Teflon filters. Redox-active volatile compounds may not be fully captured by these filters. Coarse and fine PM recovery of filters was estimated to be around 80–90%, based on other studies (Künzli et al. 2006; Shi et al. 2003a, 2006). Exact recovery is unknown and depends on the exact composition of the sampled PM.

The significance of PM oxidative potential for health at present is known only from experimental studies on biomarkers of early biological effects (Shi et al. 2003a, 2006). Almost no epidemiological studies have linked high levels of oxidative potential of particles to adverse health effects, such as respiratory or cardiovascular morbidity and mortality. In a recent panel study, Delfino et al. (2010) related oxidative potential using the alveolar macrophage ROS assay to airway and systematic inflammation in 60 elderly people. The oxidative potential effects on interleukin-6 and exhaled nitric oxide were comparable with the effects of traffic pollutants such as black carbon and particle number (Delfino et al. 2010). The use of different oxidative potential assays, difficulties in assessing exposure because the oxidative potential of PM varies substantially in time and space, and the high costs of the different assays currently hamper wide-scale use. Further studies that evaluate the relationship of oxidative potential of PM using different assays and public health are needed (Ayres et al. 2008; Künzli et al. 2006).

## Conclusions

The oxidative potential of PM_10_ collected at major streets was 3.6 times higher than that of simultaneously measured PM_10_ from urban background locations, and 6.5 times higher than PM_10_ from suburban locations. These contrasts exceeded corresponding spatial contrasts in other PM characteristics, including concentrations of transition metals such as Cu and Fe. Oxidative potential was highly correlated with soot, Ba, Cr, Cu, Fe, and Mn. The oxidative potential of PM_10_ was 4.6 times greater than the oxidative potential of PM_2.5_, measured simultaneously in a subset of filters when expressed per volume unit, and 3.1 times greater when expressed per mass unit.

Given the large contrast in oxidative potential of PM between moderately busy streets versus background locations, and some indications that oxidative potential may be more directly biologically relevant than other measures of exposure, oxidative potential may serve as a better indicator to assess and control adverse health effects of traffic-related PM.

## Supplemental Material

(41 KB) PDF
