# Supplementary material for: Contrasts in Oxidative Potential and Other Particulate Matter Characteristics Collected Near Major Streets and Background Locations
Source: Environ Health Perspect. 2011 Oct 20;120(2):185–91. doi: 10.1289/ehp.1103667 (PMC3279435; doi:10.1289/ehp.1103667)
Supplement: (41 KB) PDF [file ehp.1103667.s001.pdf]

Supplemental Material

Contrasts in Oxidative Potential and Other PM Characteristics Collected Near Major Streets and Background Locations

Hanna Boogaard, Nicole A.H. Janssen, Paul H. Fischer, Gerard P.A. Kos, Ernie P. Weijers, Flemming R. Cassee, Saskia C. van der Zee, Jeroen J. de Hartog, Bert Brunekreef, Gerard Hoek

Table of Contents

Table 1 Exact air sampling schedule

Table 2 Detailed characteristics of the different urban streets

Table 3 Median ratios streets and urban background locations vs suburban background locations

Table 4 Relation between oxidative potential and other PM characteristics - Results of mixed model analysis

Supplemental Material, Table 1 Exact air sampling schedule

		MEASUREMENT WEEK[a]												
**Cities**	**Location**	**1**	**2**	**3**	**4**	**5[b]**	**6[b]**	**7**	**8**	**9[b]**	**10[b]**	**11**	**12**	
Amsterdam	Haarlemmerweg	X			X		X		X		X		X	
	Hoofdweg	X			X		X		X		X		X	
	Urban background	X			X		X		X		X		X	
	Suburban background	X			X		X		X		X		X	Sample round A
The Hague	Stille Veerkade	X			X		X		X		X		X	
	Urban background	X			X		X		X		X		X	
	Suburban background	X			X		X		X		X		X	
Den Bosch	Brugstraat		X	X		X		X		X		X		
	Koningsweg		X	X		X		X		X		X		
	Urban background		X	X		X		X		X		X		
	Suburban background		X	X		X		X		X		X		
Tilburg	HVB		X	X		X		X		X		X		Sample round B
	Urban background		X	X		X		X		X		X		
	Suburban background		X	X		X		X		X		X		
Utrecht	Vleutenseweg		X	X		X		X		X		X		
	Weerdsingel		X	X		X		X		X		X		
	Urban background		X	X		X		X		X		X		
	Suburban background		X	X		X		X		X		X		
	Central reference	X	X	X	X	X	X	X	X	X	X	X	X	

[a]Measurement performed in 2008 and beginning of 2009; Measurement week 1: June 12 – June 19; Measurement week 2: June 24 – July 01; Measurement week 3: Sept. 1 – Sept. 08; Measurement week 4: Sept. 11 – Sept. 18; Measurement week 5: Sept. 29 – Oct. 06; Measurement week 6: Oct. 30 – Nov. 06; Measurement week 7: Nov. 10 – Nov. 17; Measurement week 8: Nov. 20 – Nov. 27.; Measurement week 9: Dec. 01 – Dec. 08; Measurement week 10: Dec. 11 – Dec. 18; Measurement week 11: Jan. 12 – Jan. 19; Measurement week 12: Jan. 22 – Jan. 29;
[b]In these measurement weeks oxidative potential of $PM_{2.5}$ was also estimated (subset).

Supplemental Material, Table 2 Detailed characteristics of the different urban streets

		**Traffic intensity [a]**	**Fraction[b]**		**Speed (km/h)**		**Road type**	**Distance to road axis (m)**
**Cities**	**Urban streets**	**per 24-h**	**Middle**	**Heavy**	**Per 24-h**	**In rush hours**		
Amsterdam	Haarlemmerweg	15 253	0.03	0.02	41	38	Adjoining buildings on one side	11
Amsterdam	Hoofdweg	9774	0.01	0.06	41	41	Adjoining buildings on two sides	16
The Hague	Stille Veerkade	17 438	0.05	0.02	34	32	Canyon[c]	11
Den Bosch	Brugstraat	17 896	0.05	0.05	32	28	Canyon[c]	9
Den Bosch	Koningsweg	17 138	0.05	0.03	46	39	Adjoining buildings on two sides	14
Tilburg	HVB	18 812	0.03	0.07	51	49	Adjoining buildings on two sides	12
Utrecht	Vleutenseweg	13 553	0.06	0.05	39	36	Adjoining buildings on two sides	15
Utrecht	Weerdsingel Wz	14 831	0.06	0.03	35	31	Adjoining buildings on one side	9

[a]Total motorized vehicles per 24-h.

[b]Fraction of traffic intensity. Middle traffic characterized as traffic with a distance between the wheel axes of 3.5 to 7 m.

[c]Narrow street with adjoining buildings on two sides. Distance between façade and road axis is smaller than 1.5 times the height of the buildings.

Supplemental Material, Table 3 Median ratios streets and urban background locations vs suburban background locations

Cities	Streets and urban background locations	·OH
Amsterdam	Haarlemmerweg	11.6
Amsterdam	Hoofdweg	4.2
Amsterdam	Urban background	1.8
The Hague	Stille Veerkade	8.7
The Hague	Urban background	2.1
Den Bosch	Brugstraat	11.6
Den Bosch	Koningsweg	8.2
Den Bosch	Urban background	3.1
Tilburg	HVB	5.3
Tilburg	Urban background	1.5
Utrecht	Vleutenseweg	2.2
Utrecht	Weerdsingel Wz	6.1
Utrecht	Urban background	1.6
	Overall ratio streets	6.5
	P-value	<0.0001
	Overall ratio urban background	1.8
	P-value	<0.0001

Supplemental Material, Table 4 Relation between oxidative potential and other PM characteristics[a] - Results of mixed model analysis

	IQR[b]	Beta[c]	Standard error	P-value	AIC[d]
**One-pollutant model**					
PM_{10}	13	-0.0100	0.0068	0.0867	232.7
$PM_{2.5}$	14	-0.0100	0.0072	0.1235	220.8
'Soot'	1.4	0.2600	0.0717	0.0005	220.7
Ba	9	0.0241	0.0105	0.0235	229.9
Cu	22	0.0368	0.0040	<0.0001	198.6
Cr	3	0.0734	0.0299	0.0163	227.8
Fe	471	0.0012	0.0002	<0.0001	226.1
Mn	7	0.0066	0.0126	0.6016	234.1
S	834	-0.0002	0.0001	0.0132	237.9
**Two-pollutant model**					
Intercept		9.2829	0.1654		181.8
Cu	22	0.0462	0.0040	<0.0001	
PM_{10}	13	-0.0335	0.0063	<0.0001	

[a] PM_{10} and $PM_{2.5}$ in $\mu g/m^3$; Elemental concentrations from PM_{10} filters in ng/m^3. 'soot' in $10^{-5}m^{-1}$. The logarithm of the PM_{10} oxidative potential was used as dependant variable.

[b] IQR = Inter quartile range

[c] To estimate percentage change per IQR of a pollutant the following formula can be used: $((e^{IQR*beta}) -1)*100$.

[d] AIC = Akaike information criterion (the lower, the better model fit)
